# Global validation of the WSES Sepsis Severity Score for patients with complicated intra-abdominal infections: a prospective multicentre study (WISS Study)

**DOI:** 10.1186/s13017-015-0055-0

**Published:** 2015-12-16

**Authors:** Massimo Sartelli, Fikri M. Abu-Zidan, Fausto Catena, Ewen A. Griffiths, Salomone Di Saverio, Raul Coimbra, Carlos A. Ordoñez, Ari Leppaniemi, Gustavo P. Fraga, Federico Coccolini, Ferdinando Agresta, Asrhaf Abbas, Saleh Abdel Kader, John Agboola, Adamu Amhed, Adesina Ajibade, Seckin Akkucuk, Bandar Alharthi, Dimitrios Anyfantakis, Goran Augustin, Gianluca Baiocchi, Miklosh Bala, Oussama Baraket, Savas Bayrak, Giovanni Bellanova, Marcelo A. Beltràn, Roberto Bini, Matthew Boal, Andrey V. Borodach, Konstantinos Bouliaris, Frederic Branger, Daniele Brunelli, Marco Catani, Asri Che Jusoh, Alain Chichom-Mefire, Gianfranco Cocorullo, Elif Colak, David Costa, Silvia Costa, Yunfeng Cui, Geanina Loredana Curca, Terry Curry, Koray Das, Samir Delibegovic, Zaza Demetrashvili, Isidoro Di Carlo, Nadezda Drozdova, Tamer El Zalabany, Mushira Abdulaziz Enani, Mario Faro, Mahir Gachabayov, Teresa Giménez Maurel, Georgios Gkiokas, Carlos Augusto Gomes, Ricardo Alessandro Teixeira Gonsaga, Gianluca Guercioni, Ali Guner, Sanjay Gupta, Sandra Gutierrez, Martin Hutan, Orestis Ioannidis, Arda Isik, Yoshimitsu Izawa, Sumita A. Jain, Mantas Jokubauskas, Aleksandar Karamarkovic, Saila Kauhanen, Robin Kaushik, Jakub Kenig, Vladimir Khokha, Jae Il Kim, Victor Kong, Renol Koshy, Avidyl Krasniqi, Ashok Kshirsagar, Zygimantas Kuliesius, Konstantinos Lasithiotakis, Pedro Leão, Jae Gil Lee, Miguel Leon, Aintzane Lizarazu Pérez, Varut Lohsiriwat, Eudaldo López-Tomassetti Fernandez, Eftychios Lostoridis, Raghuveer Mn, Piotr Major, Athanasios Marinis, Daniele Marrelli, Aleix Martinez-Perez, Sanjay Marwah, Michael McFarlane, Renato Bessa Melo, Cristian Mesina, Nick Michalopoulos, Radu Moldovanu, Ouadii Mouaqit, Akutu Munyika, Ionut Negoi, Ioannis Nikolopoulos, Gabriela Elisa Nita, Iyiade Olaoye, Abdelkarim Omari, Paola Rodríguez Ossa, Zeynep Ozkan, Ramakrishnapillai Padmakumar, Francesco Pata, Gerson Alves Pereira Junior, Jorge Pereira, Tadeja Pintar, Konstantinos Pouggouras, Vinod Prabhu, Stefano Rausei, Miran Rems, Daniel Rios-Cruz, Boris Sakakushev, Maria Luisa Sánchez de Molina, Charampolos Seretis, Vishal Shelat, Romeo Lages Simões, Giovanni Sinibaldi, Matej Skrovina, Dmitry Smirnov, Charalampos Spyropoulos, Jaan Tepp, Tugan Tezcaner, Matti Tolonen, Myftar Torba, Jan Ulrych, Mustafa Yener Uzunoglu, David van Dellen, Gabrielle H. van Ramshorst, Giorgio Vasquez, Aurélien Venara, Andras Vereczkei, Nereo Vettoretto, Nutu Vlad, Sanjay Kumar Yadav, Tonguç Utku Yilmaz, Kuo-Ching Yuan, Sanoop Koshy Zachariah, Maurice Zida, Justas Zilinskas, Luca Ansaloni

**Affiliations:** Department of Surgery, Macerata Hospital, Macerata, Italy; Department of Surgery, College of Medicine and Health Sciences, UAE University, Al-Ain, United Arab Emirates; Department of Emergency Surgery, Maggiore Hospital, Parma, Italy; General and Upper GI Surgery, Queen Elizabeth Hospital, Birmingham, UK; Department of Surgery, Maggiore Hospital, Bologna, Italy; Department of Surgery, UC San Diego Medical Center, San Diego, USA; Fundación Valle del Lili, Universidad del Valle, Cali, Colombia; Abdominal Center, University Hospital Meilahti, Helsinki, Finland; Division of Trauma Surgery, Hospital de Clinicas, School of Medical Sciences, University of Campinas, Campinas, Brazil; General and Emergency Surgery, Papa Giovanni XXIII Hospital, Bergamo, Italy; General Surgery, ULSS19 del Veneto, Adria, RO Italy; Department of Surgery, Mansoura University Hospital, Mansoura, Egypt; Department of General Surgery, Al Ain Hospital, Al-Ain City, United Arab Emirates; Department of Surgery, Kwara State General Hospital, Ilorin, Nigeria; Department of Surgery, Ahmadu Bello University Teaching Hospital Zaria, Kaduna, Nigeria; Department of Surgery, LAUTECH Teaching Hospital, Osogbo, Nigeria; Department of General Surgery, Training and Research Hospital of Mustafa Kemal University, Hatay, Turkey; Depatment of Surgery, King Fahad Medical City, Riyadh, Saudi Arabia; Primary Health Care Centre of Kissamos, Chania, Greece; Department of Surgery, University Hospital Center, Zagreb, Croatia; Clinical and Experimental Surgery, Brescia Civil Hospital, Brescia, Italy; Trauma and Acute Care Surgery Unit, Hadassah Hebrew University Medical Center, Jerusalem, Israel; Department of Surgery, Bizerte Hospital, Bizerte, Tunisia; Department of General Surgery, Istanbul Training and Research Hospital, Istanbul, Turkey; Surgical II Division, S. Chiara Hospital, Trento, Italy; Department of General Surgery, Hospital San Juan de Dios de La Serena, La Serena, Chile; Department of General and Emergency Surgery, SG Bosco Hospital, Turin, Italy; Emergency Surgery Department, 1st Municipal Hospital, Novosibirsk State Medical University, Novosibirsk, Russian Federation; Department of Surgery, University Hospital of Larissa, Larissa, Greece; Visceral Surgery, CHU, Angers, France; Chirurgia Generale, Ospedale di Città di Castello, Città di Castello, Italy; Department of Emergency Surgery, Umberto I Hospital, “La Sapienza” University, Rome, Italy; Department of Surgery, Kuala Krai Hospital, Kelantan, Malaysia; Department of Surgery, Regional Hospital, Limbe, Cameroon; General and Emergency Surgery, Policlinico Paolo Giaccone, Palermo, Italy; Department of General Surgery, Samsun Education and Research Hospital, Samsun, Turkey; Department of General and Digestive Tract Surgery, Alicante University General Hospital, Alicante, Spain; Department of Surgery, CHVNG/E, EPE, Vila Nova de Gaia, Portugal; Department of Surgery, Tianjin Nankai Hospital, Tianjin, China; Department of General Surgery, Emergency Municipal Hospital Pascani, Pascani, Iasi Romania; Department of Surgery, Numune Training and Research Hospital, Adana, Turkey; Department of Surgery, University Clinical Center, Tuzla, Bosnia and Herzegovina; Department General Surgery, Kipshidze Central University Hospital, Tbilisi, Georgia; Department of Surgery, Hamad General Hospital, Doha, Qatar; Department of Surgery, Riga East Clinical University Hospital, Riga, Latvia; Department of Surgery, Bahrain Defence Force Hospital, Manama, Bahrain; King Fahad Medical City, Riyadh, Saudi Arabia; Division of General and Emergency Surgery, Hospital Estadual Mario Covas, ABC School of Medicine, Santo André, Brazil; Department of Surgery 1, Vladimir City Clinical Hospital of Emergency Medicine, Vladimir, Russian Federation; Cirugía General y Digestiva, Hospital Universitario Miguel Servet, Zaragoza, Spain; 2nd Department of Surgery, Aretaieio University Hospital, Athens, Greece; Department of Surgery, Hospital Universitário Terezinha de Jesus, Faculdade de Ciências Médicas e da Saúde de Juiz de Fora, Juiz de Fora, Brazil; Department of Surgery, Hospital Escola Padre Albino, Catanduva, Brazil; Department of Surgery, Ascoli Piceno Hospital, Ascoli Piceno, Italy; Department of General Surgery, Trabzon Kanuni Training and Research Hospital, Trabzon, Turkey; Department of Surgery, Government Medical College and Hospital, Chandigarh, India; Hospital Universitario del Valle, Universidad del Valle, Cali, Colombia; 2nd Surgical Department of Medical Faculty Comenius University, University Hospital Bratislava, Bratislava, Slovakia; 2nd Surgical Department, General Hospital of Kavala, Kavala, Greece; Department of Surgery, Mengucek Gazi Training Research Hospital, Erzincan, Turkey; Department of Emergency and Critical Care Medicine, Jichi Medical University, Shimotsuke, Japan; Department of Surgery, S M S Hospital, Jaipur, India; Department of Surgery, Hospital of Lithuanian University of Health Sciences, Kaunas, Lithuania; Clinic for Emergency Surgery, Medical Faculty University of Belgrade, Belgrade, Serbia; Division Digestive Surgery and Urology, Turku University Hospital, Turku, Finland; 3rd Department of General Surgery, Jagiellonian Univeristy Collegium Medium, Kraków, Poland; Department of Emergency Surgery, City Hospital, Mozyr, Belarus; Department of Surgery, Ilsan Paik Hospital, Inje University College of Medicine, Goyang, Republic of Korea; Department of Surgery, Edendale Hospital, Pietermaritzburg, South Africa; Department of Surgery, University Clinical Center of Kosovo, Pristina, Kosovo; Department of General Surgery, Krishna Hospital, Karad, India; Department of General Surgery, Republican Vilnius University Hospital, Vilnius, Lithuania; Department of Surgery, York Teaching Hospital NHS Foundation Trust, York, UK; General Surgery/Coloretal Unit, Braga Hospital, Life and Health Sciences Research Institute (ICVS), School of Health Sciences, University of Minho, Braga, Portugal; Department of Surgery, Yonsei University College of Medicine, Seoul, South Korea; Department of Surgery, Hospital La Paz, Madrid, Spain; Cirugía de Urgencias, Hospital Universitario Donostia, Donostia, Spain; Department of Surgery, Faculty of Medicine Siriraj Hospital, Mahidol University, Bangkok, Thailand; Department of Surgery, Insular University Hospital of Gran Canaria, Las Palmas, Spain; 1st Department of Surgery, Kavala General Hospital, Kavala, Greece; Department of General Surgery, Mysore Medical College and Research Institute, Government Medical College Hospital Mysore, Mysore, India; 2nd Department of Surgery, Jagiellonian University Medical College, Krakow, Poland; First Department of Surgery, Tzaneio Hospital, Piraeus, Greece; Department of General Surgery and Surgical Oncology, Le Scotte Hospital, Siena, Italy; Department of Surgery, University Hospital, Valencia, Spain; Department of Surgery, Post-Graduate Institute of Medical Sciences, Rohtak, India; Department of Surgery, Radiology, University Hospital of the West Indies, Kingston, Jamaica; General Surgery Department, Centro Hospitalar de São João, Porto, Portugal; Second Surgical Clinic, Emergency Hospital of Craiova, Craiova, Romania; 3rd Department of Surgery, Haepa University Hospital, Thessaloniki, Greece; Department of Surgery, CH Armentieres, Arras, France; Surgery Department, University Hospital Hassan II, Fez, Morocco; Department of Surgery, Onandjokwe Hospital, Ondangwa, Namibia; Department of Surgery, Emergency Hospital of Bucharest, Bucharest, Romania; Department of General Surgery, Lewisham & Greenwich NHS Trust, London, UK; Department of Surgery, University of Ilorin Teaching Hospital, Ilorin, Nigeria; Department of Surgery, King Abdalla University Hospital, Irbid, Jordan; Department of Surgery, Elazig Training and Research Hospital, Elazig, Turkey; Department of Laparoscopic and Metabolic Surgery, Sunrise Hospital, Kochi, India; Department of Surgery, Sant’Antonio Abate Hospital, Gallarate, Italy; Division of Emergency and Trauma Surgery, Ribeirão Preto Medical School, Ribeirão Preto, Brazil; Surgery 1 Unit, Centro Hospitalar Tondela Viseu, Viseu, Portugal; Department of Surgery, UMC Ljubljana, Ljubljana, Slovenia; Department of Surgery, Bharati Medical College and Hospital, Sangli, India; Department of Surgery, Insubria University Hospital, Varese, Italy; Abdominal and General Surgery Department, General Hospital Jesenice, Jesenice, Slovenia; Department of Surgery, Hospital de Alta Especialidad de Veracruz, Veracruz, Mexico; General Surgery Department, Medical University, University Hospital St George, Plovdiv, Bulgaria; Department of Surgery, Fundación Jimenez Díaz, Madrid, Spain; Department of Surgery, Good Hope Hospital, Heart of England NHS Foundation Trust, Birmingham, UK; Department of General Surgery, Tan Tock Seng Hospital, Novena, Singapore; Departement of Surgery, Fatabenefratelli Isola Tiberina Hspital, Rome, Italy; Department of Surgery, Hospital and Comprehensive Cancer Centre Novy Jicin, Novy Jicin, Czech Republic; Department of General Surgery, Clinical Hospital at Chelyabinsk Station of OJSC “Russian Railroads”, Chelyabinsk, Russian Federation; 3th Department of Surgery, Iaso General Hospital, Athens, Greece; Department of Surgery, North Estonia Medical Center, Tallin, Estonia; Department of Surgery, Baskent University Ankara Hospital, Ankara, Turkey; General Surgery Service, Trauma University Hospital, Tirana, Albania; 1st Department of Surgery - Department of Abdominal, Thoracic Surgery and Traumatology, General University Hospital, Prague, Czech Republic; Department of General Surgery, Sakarya Teaching and Research Hospital, Sakarya, Turkey; Department of Renal and Pancreas Transplantation, Manchester Royal Infirmary, Manchester, UK; Department of Surgery, Red Cross Hospital, Beverwijk, Netherlands; Emergency Surgery, Arcispedale S.Anna Azienda Ospedaliero-Universitaria di Ferrara, Ferrara, Italy; Department of Surgery, Medical School University Pecs, Pecs, Hungary; Department of Surgery, Montichiari Hospital, Ospedali Civili Brescia, Brescia, Italy; 1st Surgical Clinic, St. Spiridon Hospital, Iasi, Romania; Department of Surgery, Rajendra Institute of Medical Sciences, Ranchi, India; Department of Surgery, Kocaeli University Training and Research Hospital, Kocaeli, Turkey; Trauma and Emergency Surgery Department, Chang Gung Memorial Hospital, Taoyuan City, Taiwan; Department of Surgery, MOSC Medical College Kolenchery, Cochin, India; General and Digestive Surgery Department, Teaching Hospital Yalgado Ouedraogo, Ouagadougou, Burkina Faso

**Keywords:** Intra-abdominal, Infections, Sepsis, Septic shock

## Abstract

**Background:**

To validate a new practical Sepsis Severity Score for patients with complicated intra-abdominal infections (cIAIs) including the clinical conditions at the admission (severe sepsis/septic shock), the origin of the cIAIs, the delay in source control, the setting of acquisition and any risk factors such as age and immunosuppression.

**Methods:**

The WISS study (WSES cIAIs Score Study) is a multicenter observational study underwent in 132 medical institutions worldwide during a four-month study period (October 2014-February 2015). Four thousand five hundred thirty-three patients with a mean age of 51.2 years (range 18–99) were enrolled in the WISS study.

**Results:**

Univariate analysis has shown that all factors that were previously included in the WSES Sepsis Severity Score were highly statistically significant between those who died and those who survived (*p* < 0.0001). The multivariate logistic regression model was highly significant (*p* < 0.0001, R2 = 0.54) and showed that all these factors were independent in predicting mortality of sepsis. Receiver Operator Curve has shown that the WSES Severity Sepsis Score had an excellent prediction for mortality. A score above 5.5 was the best predictor of mortality having a sensitivity of 89.2 %, a specificity of 83.5 % and a positive likelihood ratio of 5.4.

**Conclusions:**

WSES Sepsis Severity Score for patients with complicated Intra-abdominal infections can be used on global level. It has shown high sensitivity, specificity, and likelihood ratio that may help us in making clinical decisions.

## Background

Intra-abdominal infections (IAIs) include several different pathological conditions [1] and are usually classified into uncomplicated and complicated. In complicated IAIs (cIAIs), the infectious process extends beyond the organ, and causes either localized peritonitis or diffuse peritonitis. The treatment of patients with complicated intra-abdominal infections involves both source control and antibiotic therapy. Complicated IAIs are an important cause of morbidity and may be associated with poor prognosis. However the term “complicated intra-abdominal infections” describes a wide heterogeneity of patient populations, making it difficult to suggest a general treatment regimen and stressing the need of an individualized approach to decision making.

Early prognostic evaluation of complicated intra-abdominal infections is crucial to assess the severity and decide the aggressiveness of treatment. Many factors influencing the prognosis of patients with cIAIs have been described, including advanced age, poor nutrition, pre-existing diseases, immunosuppression, extended peritonitis, occurrence of septic shock, poor source control, organ failures, prolonged hospitalization before therapy, and infection with nosocomial pathogens [2–10].

Recently the World Society of Emergency Surgery (WSES) designed a global prospective observational study (CIAOW Study) [11, 12]. All the risk factors for occurrence of death during hospitalization were evaluated and then discussed with an international panel of experts. The most significant variables, adjusted to clinical criteria, were used to create a severity score for patients with cIAIs including the clinical conditions at admission (severe sepsis/septic shock), the origin of the cIAIs, the delay in source control, the setting of acquisition and any risk factors such as age and immunosuppression (Appendix).

There may be different causes of sepsis, health care standards, and differences in underlying health status, economical differences that make prediction of sepsis on global level difficult. The WSES addressed this issue in the present study which aims to validate a previous score on a global level.

## Methods

### Ethical statement

The study met the standards outlined in the Declaration of Helsinki and Good Epidemiological Practices. This study did not change or modify the laboratory or clinical practices of each centre and differences of practices were kept as they are. The data collection was anonymous and identifiable patient information was not submitted.

Individual researchers were responsible for complying with local ethical standards and hospital registration of the study.

### Study population

This multicenter observational study was run in 132 medical institutions from 54 countries worldwide during a four-month period (October 2014-February 2015). Inclusion criteria were patients older than 18 years with complicated intra-abdominal sepsis (cIAIs) who had surgical management or interventional radiological drainage. cIAIs was defined as an infectious process that proceeded beyond the organ, and caused either localized peritonitis/abscess or diffuse peritonitis [13]. Patients who were younger than 18 years, or those who had pancreatitis, or primary peritonitis were excluded from the study. Severe sepsis was defined as sepsis-induced tissue hypoperfusion or organ dysfunction (any of the following thought to be due to the infection): hypotension (<90/60 or MAP < 65), lactate above upper limits laboratory normal, Urine output < 0.5 mL/kg/h for more than 2 h despite adequate fluid resuscitation, Creatinine > 2.0 mg/dL (176.8 μmol/L), Bilirubin > 2 mg/dL (34.2 μmol/L), Platelet count < 100,000 μL, Coagulopathy (international normalized ratio > 1.5), Acute lung injury with Pao2/Fio2 < 250 in the absence of pneumonia as infection source. Septic shock was defined as severe sepsis associated with refractory hypotension (BP < 90/60) despite adequate fluid resuscitation [14].

WSES Sepsis Severity Score for patients with complicated Intra-abdominal infections is shown in Appendix**.**

### Data monitoring and collection

The study was monitored by the coordination center, which investigated and verified missing or unclear data submitted to the central database. This study was performed under the direct supervision of the Board of Directors of WSES. In each centre, the coordinator collected and compiled data in an online case report system. Data were entered directly through a web-based computerized database. Data were entered either by a drop menu for categorical data like the source of infection or numbers for continuous variables such as age. Data collected included demographic data of the patient and disease characteristics, demographical data, type of infection (community- or healthcare-acquired), severity criteria and origin of infection and surgical procedures performed.

### Statistical analysis

Sepsis status was coded as ordinal data for testing the logistic regression (not for scoring) as follows: no sepsis = 0, sepsis = 2, severe sepsis = 3, septic shock = 4). The source of sepsis was analysed as categorical data in the logistic regression, and the age as continuous data, while healthcare associated infection, delay in management, and immunosuppression as binomial data. The variables used in this scoring system in the patients who survived and those who died were compared using univariate analysis. This included Fisher’s exact test or Pearson Chi-Square as appropriate for categorical data and Mann–Whitney U-test for continuous or ordinal data. Significant factors were then entered into a direct logistic regression model. A p value of ≤ 0.05 was considered significant. Data were analyzed with PASW Statistics 21, SPSS Inc, USA.

## Results

Four thousand six hundred fifty-two cases were collected in the online case report system. One hundred twenty-nine cases did not meet the inclusion criteria. Four thousand five hundred thirty-three patients with a mean age of 51.2 years (range 18–99) were enrolled in the WISS study. One thousand nine hundred thirty-five patients (42.7 %) were women and 2598 (57.3 %) were men.

Among these patients, 3966 (87.5 %) were affected by community-acquired IAIs while the remaining 567 (12.5 %) suffered from healthcare-associated infections. One thousand six hundred twenty-seven patients (35.9 %) were affected by generalized peritonitis while 2906 (64.1 %) suffered from localized peritonitis or abscesses. Seven hundred ninety-one patients (17.4 %) were admitted in critical condition (severe sepsis/septic shock). The various sources of infection are outlined in Table 1. The most frequent source of infection was acute appendicitis; 1553 cases (34.2 %) involved complicated appendicitis.

**Table 1:** Source of infection in 4553 patients from 132 hospitals worldwide (15 October 2014–15 February 2015)

Source of infection	Number (%)
Appendicitis	1553 (34.2 %)
Cholecystitis	837 (18.5 %)
Post-operative	387 (8.5 %)
Colonic non diverticular perforation	269 (5.9 %)
Gastro-duodenal perforations	498 (11 %)
Diverticulitis	234 (5.2 %)
Small bowel perforation	243 (5.4 %)
Others	348 (7.7 %)
PID	50 (1.1 %)
Post traumatic perforation	114 (2.5 %)
Missing	
Total	4553 (100 %)

*PID* pelvic inflammatory disease

The overall mortality rate was 9.2 % (416/4533).

Table 2 shows the univariate analysis comparing patients with complicated intra-abdominal infection who survived and those who died. The analysis shows that all factors included in the Sepsis Severity Score were highly significantly different between those who died and those who survived (*p* < 0.0001 in all variables). Accordingly all factors were entered into a direct logistic regression model (Table 3). The direct logistic regression model was highly significant (*p* < 0.0001, R2 = 0.54) and showed that all factors included in the Sepsis Severity Score were significant independent predictors of mortality. Accordingly the ability of the score to predict mortality was tested by a direct logistic regression which is shown in Table 4. Again, this model using only the sepsis severity score was highly significant (*p* < 0.0001, R2 = 0.5). The odds of death increased by 0.78 by an increase on one score which is remarkable.

**Table 2:** Univariate analysis of patients with complicated intra-abdominal infection comparing patients who survived (*n* = 4117) and patient who died (*n* = 416)

Variable	Survided (%) *n* = 4117	Died (%) *n* = 416	*p* value
Sepsis status			<0.0001
No sepsis	1914 (46.5 %)	23 (5.5 %)	
Sepsis	1725 (41.9 %)	80 (19.2 %)	
Severe sepsis	404 (9.8 %)	157 (37.7 %)	
Septic shock	74 (1.8 %)	156 (37.5 %)	
Healthcare associated infection	433 (10.5 %)	134 (32.2 %)	<0.0001
Source of infection			<0.0001
Appendicitis	1536 (37.3 %)	17 (4.1 %)	
Cholecystitis	809 (19.7 %)	28 (6.7 %)	
Colonic non diverticular perforation	204 (5 %)	65 (15.6 %)	
Diverticulitis	203 (4.9 %)	31 (7.5 %)	
Gastro-duodenal perforation	431 (10.5 %)	67 (16.2 %)	
PID	50 (1.2 %)	0 (0)	
Postoperative	415 (10.1 %)	86 (20.7 %)	
Small bowel perforation	174 (4.2 %)	69 (16.6 %)	
Post-traumatic	104 (2.5 %)	10 (2.4 %)	
Others	259 (6.3 %)	53 (12.7 %)	
Delay in source control	2015 (48.9 %)	341 (82 %)	<0.0001
Median age years (range)	48 (18–97)	79 (18–99)	<0.0001
Immunosuppresion	292 (7.1)	120 (28.8 %)	<0.0001
Sepsis severity score	3 (0–17)	10 (0–17)	<0.0001

Data presented as median range or number percentage as appropriate

*PID* pelvic inflammatory disease

*p* value = Fisher’s exact test, Pearson Chi-Square, or Mann Whitney U test as appropriate

**Table 3:** Direct logistic regression model with factors affecting mortality of patients complicated intra-abdominal infection, global study of 132 centres, (*n* = 4553)

Score variable	B	S.E.	Wald test	*P* value	OR	OR 95 % C.I.	
						Lower	Upper
Sepsis status	1.57	0.08	365.59	<0.0001	4.81	4.09	5.65
Setting of infection acquisition	0.6	0.18	10.49	0.001	1.81	1.27	2.6
Source of infection^a^			59.38	<0.0001			
Colonic non-diverticulical perforation	−0.26	0.27	0.97	0.33	0.77	0.46	1.3
Diverticulitis diffuse peritonitis	−0.26	0.34	0.51	0.48	0.78	0.40	1.54
Postoperative diffuse peritonitis	−0.005	0.29	0	0.99	1.00	0.56	1.76
Remaining sources	−1.2	0.21	32.47	<0.0001	0.30	0.20	0.46
Delay in management	1.47	0.17	78.53	<0.0001	4.33	3.13	5.99
Age	0.04	0.004	103.58	<0.0001	1.04	1.04	1.05
Immunosuppression	1.24	0.17	55.79	<0.0001	3.46	2.5	4.79
Constant	−7.52	0.41	342.24	<0.0001	0.001		

*OR* odds ratio

^a^Compared with small bowel perforation

**Table 4:** Direct logistic regression model showing the ability of WSES Sepsis Severity Score in predicting mortality of patients complicated intra-abdominal infection, global study of 132 centres, (*n* = 4553)

Variable	B	S.E.	Wald	*P* value	OR	OR 95 % C.I.	
						Lower	Upper
WSESSCORE	0.58	0.02	639.59	<0.0001	1.784	1.706	1.866
Constant	−5.79	0.19	958.74	<0.0001	.003		

*OR* odds ratio

**Fig. 1:**
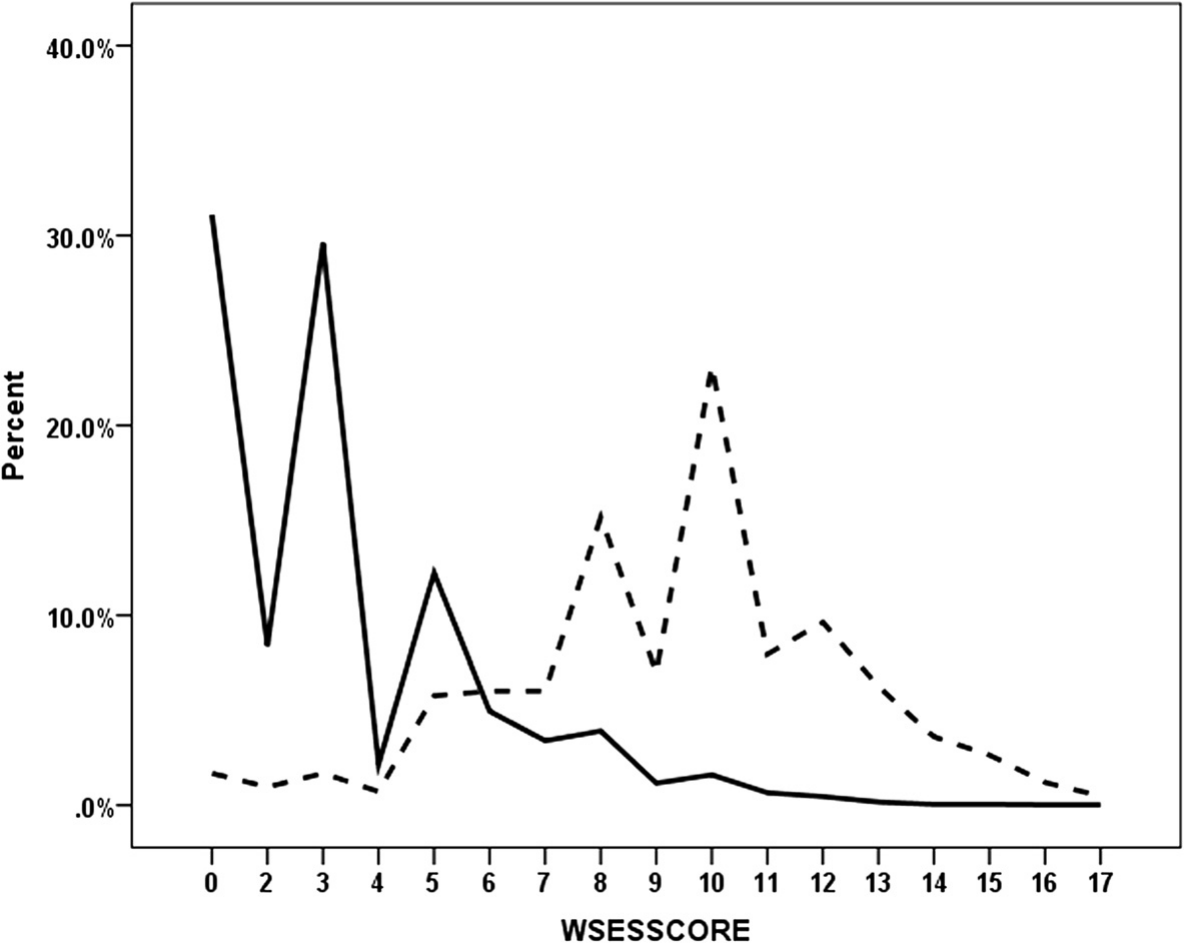
Distribution of the percentile WSES Sepsis Severity Score of complicated intra-abdominal infection patients for those who survived (*solid line*) (*n* = 4117) and those who died (*interrupted line*) (*n* = 416)

**Fig. 2:**
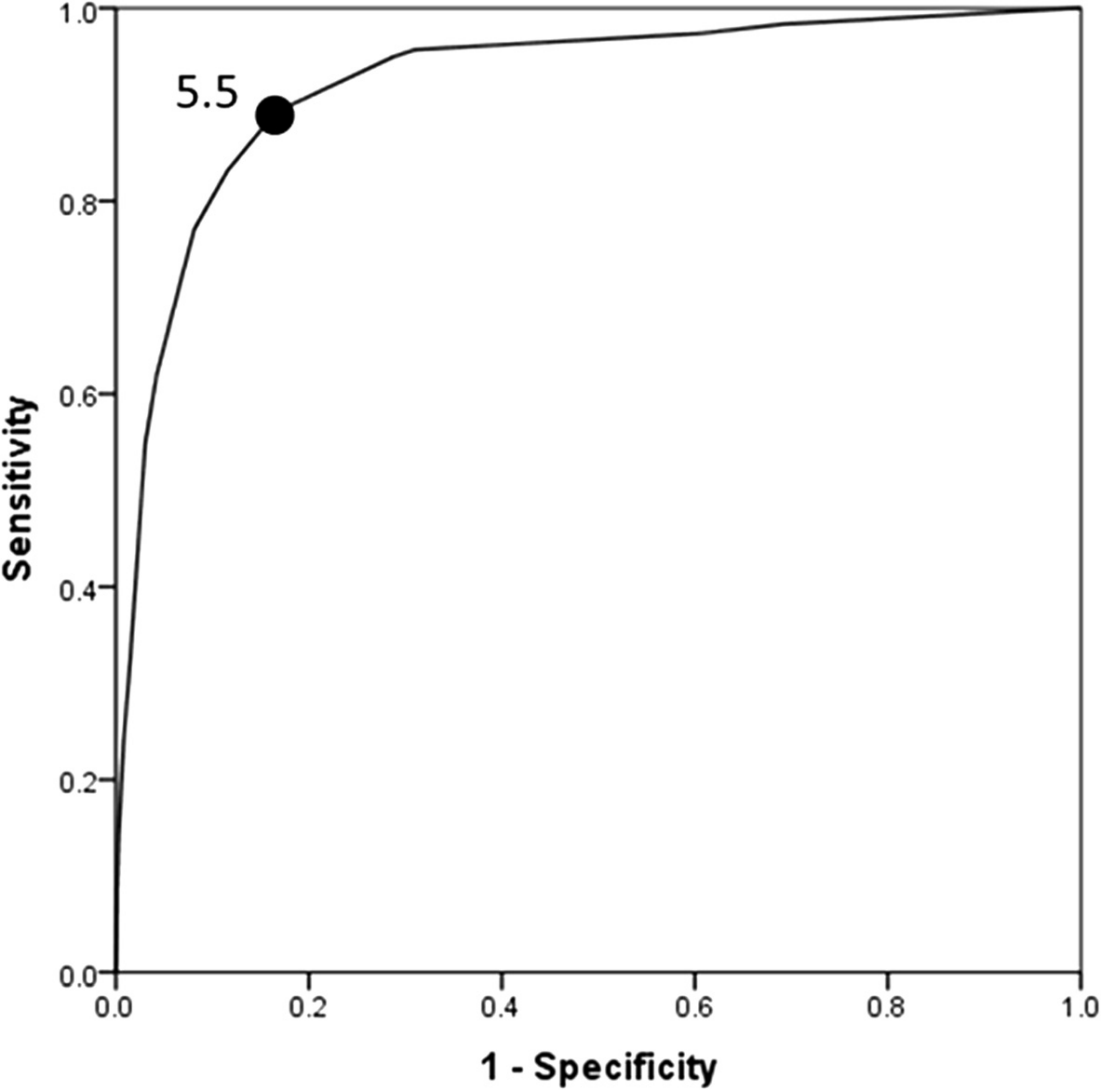
Receiver operating characteristic curve for the best WSES Sepsis Severity Score that predicted mortality in patients having complicated intra-abdominal infection, global study of 132 centres, (*n* = 4553)

Figure 1 shows that WSES Sepsis Severity Score had a very good ability of distinguishing those who survived from those who died. The overall mortality rate was 9.2 % (416/4533). This was 0.63 % for those who had a score of 0–3, 6.3 % for those who had a score of 4–6, and 41.7 % for those who had a score of ≥ 7. The receiver operating characteristic curve showed that the best cutoff point for predicting mortality was a Sepsis Severity Score. 5.5 was the best predictor of mortality having a sensitivity of 89.2 %, a specificity of 83.5 % and a positive likelihood ratio of 5.4 (Fig. 2).

## Discussion

Complicated intra-abdominal infections remain an important source of patient morbidity and may be frequently associated with poor clinical prognosis. Treatment of patients with cIAIs, has been usually described to achieve satisfactory results if adequate management is established [15]. However, results from published clinical trials may not be representative of the true morbidity and mortality rates of such severe infections. First of all, patients who have perforated appendicitis are usually over-represented in clinical trials. Furthermore patients with intra-abdominal infection enrolled in clinical trials have often an increased likelihood of cure and survival. In fact the trial eligibility criteria usually restrict the inclusion of patients with co-morbid diseases that would increase the death rate of patients with intra-abdominal infections [16]. In the WISS study we enrolled all the patients older than 18 years old with complicated intra-abdominal infections in the study-period and the overall mortality rate was 9.2 % (416/4533). Stratification of the patient’s risk is essential in order to optimize the treatment plan. Patients with intra-abdominal infections are generally classified into low risk and high risk. “High risk” is generally intended to describe patients with a high risk for treatment failure and mortality. In high risk patients the increased mortality associated with inappropriate management cannot be reversed by subsequent modifications. Therefore early prognostic evaluation of complicated intra-abdominal infections is important to assess the severity and decide the aggressiveness of treatment.

Scoring systems can be roughly divided into two groups: disease-independent scores for evaluation of serious patients requiring care in the intensive care unit (ICU) such as APACHE II and Simplified Acute Physiology Score (SAPS II) and peritonitis-specific scores such as Mannheim Peritonitis Index (MPI) [17].

Although considered a good marker, APACHE II value in peritonitis has been questioned because of the difficulty of the APACHE II to evaluate interventions despite the fact that interventions might significantly alter many of the physiological variables. Moreover it requires appropriate software to be calculated [18].

The MPI is specific for peritonitis and easy to calculate. MPI was designed by Wacha and Linder in 1983 [19]. It was based on a retrospective analysis of data from 1253 patients with peritonitis. Among 20 possible risk factors, only 8 proved to be of prognostic relevance and were entered into the Mannheim Peritonitis Index, classified according to their predictive power. After 30 years, identifying a new clinical score to assess the severity the cIAIS would be clinically relevant in order to modulate the aggressiveness of treatment according the type of infection and the clinical characteristics of the patients.

WSES Sepsis Severity Score is a new practical clinical severity score for patients with complicated intra-abdominal infections. It is specific for cIAIs and easy to calculate, even during surgery. It may be relevant in order to modulate the aggressiveness of treatment particularly in higher risk patients.

The score is illustrated in Appendix. The statistical analysis shows that the sepsis severity score has a very good ability of distinguishing those who survived from those who died. The overall mortality was 0.63 % for those who had a score of 0–3, 6.3 % for those who had a score of 4–6, 41.7 % for those who had a score of ≥ 7. In patients who had a score of ≥ 9 the mortality rate was 55.5 %, those who had a score of ≥ 11 the mortality rate was 68.2 % and those who had a score ≥ 13 the mortality rate was 80.9 %.

## Conclusions

Given the sweeping geographical distribution of the participating medical centers, WSES Sepsis Severity Score for patients with complicated Intra-abdominal infections can be used on global level. It has shown high sensitivity, specificity, and likelihood ratio that may help us in making clinical decisions.

## Appendix

**Table 5:** WSES sepsis severity score for patients with complicated Intra-abdominal infections (Range: 0–18)

Clinical condition at the admission	
• Severe sepsis (acute organ dysfunction) at the admission	3 score
• Septic shock (acute circulatory failure characterized by persistent arterial hypotension. It always requires vasopressor agents) at the admission	5 score
Setting of acquisition	
• Healthcare associated infection	2 score
Origin of the IAIs	
• Colonic non-diverticular perforation peritonitis	2 score
• Small bowel perforation peritonitis	3 score
• Diverticular diffuse peritonitis	2 score
• Post-operative diffuse peritonitis	2 score
Delay in source control	
• Delayed initial intervention [Preoperative duration of peritonitis (localized or diffuse) > 24 h)]	3 score
Risk factors	
• Age>70	2 score
• Immunosuppression (chronic glucocorticoids, immunosuppresant agents, chemotherapy, lymphatic diseases, virus)	3 score
